# Transcranial magnetic stimulation for the treatment of chronic low back pain: a narrative review

**DOI:** 10.3389/fpain.2023.1092158

**Published:** 2023-05-05

**Authors:** Camille Olechowski, Maricar Gener, Rohit Aiyer, Nicholas Mischel

**Affiliations:** ^1^ Department of Psychiatry and Behavioral Neurosciences, Wayne State University School of Medicine, Detroit, MI, United States; ^2^ Department of Psychiatry, Michigan State University, East Lansing MI, United States; ^3^ Department of Psychiatry, Richmond Interventional Pain Management PC, Staten Island, NY, United States

**Keywords:** chronic lower back pain, transcranial magnetic stimulation (TMS), low back pain and TMS, low back pain, neuromodulation

## Abstract

**Background:**

Chronic low back pain is a debilitating condition that impacts millions of individuals around the world, and also has an enormous economic impact. The impact of chronic pain does not only involve physical health, but can also play a detrimental role in a patient's mental health. Consequently, it is critical to approach these patients with multimodal management. Initially, a treatment plan which includes medications, psychotherapy, physical therapy, and invasive interventions can be utilized for chronic back pain. However, many patients experience refractory low back pain to these initial treatments, which can result in non-resolving chronic pain. As a result, many new interventions have been developed in recent years to treat refractory low back pain, including non-invasive transcranial magnetic stimulation. In recent years, there has been some limited and preliminary evidence for the treatment of chronic low back pain with transcranial magnetic stimulation, as further investigation on this intervention is warranted. After reviewing analytically high impact studies, our objective is to provide a narrative review of the treatment of chronic low back pain with repetitive transcranial magnetic stimulation (rTMS).

**Methods:**

We performed a comprehensive database search on PubMed, Embase, PsychInfo, Web of Science, and CINAHL for literature that pertains to the treatment of chronic low back pain with transcranial magnetic stimulation using these terms: “Chronic Low Back Pain and Transcranial Magnetic Stimulation”, “Low Back Pain and Transcranial Magnetic Stimulation”, “Chronic Back Pain and Transcranial Magnetic Stimulation”, “Chronic Low Back Pain and TMS”, “Low Back Pain and TMS”, and “Chronic Back Pain and TMS”. We aim to provide a narrative review of the role of rTMS in CLBP.

**Results:**

Initial search results from September to November 2021 using the above-mentioned search criteria included 458 articles, of which 164 duplicates were removed and 280 were further excluded by a three-person (CO, NM and RA) screening process. Articles were further filtered based on various exclusion and inclusion criteria. The resulting 6 studies are discussed.

**Discussion:**

The studies reviewed suggest the potential benefit in chronic lower back pain symptoms after various rTMS protocols and sites of stimulation. However, the included studies are not without issues in design for example: not randomized, not blinded, or have small sample size. This review highlights the need for scaled, better controlled research studies and standardization of treatment protocols to determine if rTMS for chronic lower back pain will be accepted as a standard treatment option for patients with chronic lower back pain symptoms.

Lower back pain has been proposed to affect over 50%–80% of adults at some point in their lives (1). First line treatment modalities include medications, psychotherapy, physical therapy, and minimally invasive interventions. Despite the established benefit of these treatments, many patients experience refractory low back pain following these initial treatments, which can result in intractable chronic pain. Chronic lower back pain (CLBP) is defined in the literature as symptoms lasting for over 3 months. Chronic pain is well established as a physically debilitating condition, and it also is detrimental to many aspects of mental health. It also has a major economic impact; it has been estimated that chronic pain costs the United States up to $635 billion a year (2).

In the literature, chronic pain is often associated with increased rates of depression and anxiety, and it is often associated with interruptions in sleep. Across different studies the prevalence for congruent major depression in patients with pain ranges from 5% to 85% and that the prevalence of pain symptoms in patients with depression ranges from 15% to 100% (3). It has also been reported that 60% of patients with chronic pain meet criteria for co-morbid anxiety disorders (4). It has also been estimated that 50%–80% of people living with chronic pain experience sleep disturbances (5). As evident above chronic pain can be debilitating in many aspects of a person's life not just the disability associated directly with pain symptoms.

One of the newer interventions to emerge over the last decade for refractory pain symptoms is the use of non-invasive repetitive transcranial magnetic stimulation (rTMS). There have been several preliminary clinical studies showing improvement in pain symptoms in conditions such as fibromyalgia, complex regional pain syndrome and chronic low back pain; however, there does not yet appear to be a standard treatment protocol for the use of rTMS in the treatment of chronic low back pain. The most common protocol in the literature for targeting pain symptoms involve either targeting the DLPFC, or the primary motor cortex (M1), usually with high frequency bursts at over 80% of resting motor threshold (6). In this review we aim to provide a comprehensive narrative review of the current published literature regarding the use of rTMS for the treatment of chronic lower back pain.

The mechanism of how rTMS works to alleviate pain symptoms is not well delineated. In general, rTMS works by changing electrical activity in different brain structures through a magnetic pulse, which when applied at a certain frequency and duration, either increase or decrease firing of neurons at the site of stimulation. Different studies have suggested that stimulation of structures such as the DLPFC or motor strip results in changes in activity in other neurological structures in the pain processing pathways (6). One theory is that an increase in activity in the DLPFC decreases activity in structures in the brain that are “over active” in different pain states. This top down control of pain processing symptoms has been supported by a study by Taylor et al. (7) that demonstrated that healthy volunteers with laboratory induced pain states reported analgesic effects with rTMS application. In addition, this study found that when naloxone an opioid inhibitor is given in conjunction with rTMS in healthy controls with laboratory induced pain symptoms, rTMS did not provide analgesic relief. This suggests that rTMS may work to alleviate pain by harnessing the brains endogenous opioid system.

We performed a comprehensive database search on PubMed, Embase, PsychInfo, Web of Science, and CINAHL for literature that pertains to the treatment of chronic low back pain with transcranial magnetic stimulation using the following terms: “Chronic Low Back Pain and Transcranial Magnetic Stimulation”, “Low Back Pain and Transcranial Magnetic Stimulation”, “Chronic Back Pain and Transcranial Magnetic Stimulation”, “Chronic Low Back Pain and TMS”, “Low Back Pain and TMS”, and “Chronic Back Pain and TMS”.

Initial search results from September to November 2021 using the above mentioned search criteria included 458 articles, of which 164 duplicates were removed and 280 were further excluded by a three person (CO, NM and RA) screening process of reviewing study abstracts by checking intervention for use of rTMS, patient population (adults over 18), and details of study design (peer reviewed publications). Studies that were excluded included those not that were not related to CLBP population specifically, review articles, studies not using rTMS, mechanistic studies and animal studies. From this initial screening, 14 studies remained, from which an additional 8 studies were further excluded after reading full text of the articles because of patient population not specific to chronic lower back pain and wrong intervention eg. peripheral magnetic stimulation versus transcranial magnetic stimulation, site of stimulation and protocol not provided. A further study by Yates et al. (8) was excluded due to only the abstract being available and it being presented as a small (*n* = 2) case study design. These final remaining 6 studies are presented below.

Most studies of rTMS in the treatment of chronic pain involved targeting the left primary motor cortex (M1). In a study by Ambriz-Tututi et al. (9), 84 subjects with CLBP received either rTMS targeted to the left primary sensory-motor cortex (*n* = 44), sham TMS (*n* = 12), or physical therapy which consisted of an application of a TENS unit to the periphery (PT) (*n* = 26). In this study, pain scores (Visual Analogue Scale (VAS), McGill Pain Questionnaire (SF-MPQ)) were significantly improved in the rTMS group compared to the sham and PT groups. The study design included 5 daily sessions of rTMS [10 biphasic pulses at 95% resting motor threshold (RMT) at 20 Hz with intertrain interval of 28 s], followed by repeat sessions at weeks 3, 4, 6, 8, 12, 20, 28 and 36. A significant reduction in pain symptoms was established from week 1 on in the rTMS treatment group compared to baseline. Interestingly, patients initially receiving the sham TMS were subsequently given rTMS at the end of the study protocol (post week 36) in a cross-over design to account for placebo effect. Significant improvement in pain symptoms in the original sham group was also observed following rTMS treatment. This study was an open label design and it cannot be ruled out that knowledge of which treatment they were receiving did not influence participated reports of pain, however results found in the cross over design do help control somewhat for the placebo affect although in the future a double blind design would be ideal to determine if the strength of the effect size is still the same. Nonetheless, this pioneering study highlights the potential of rTMS as a valuable treatment for patients with CLBP.

Preliminary results published by Mavromatis et al. (10) from a study of 10 subjects comparing inhibitory (*n* = 5) or excitatory stimulation of the M1 cortex (*n* = 5) (vs. sham) with combined physical therapy, and peripheral nerve stimulation of trunk muscles in CLBP in both groups, suggest that both paradigms of stimulation result in improved pain scores, and reduced disability at 1 month post-intervention. Continuous inhibitory stimulation appears to result in changes in excitability of the M1 cortex, whereas excitatory stimulation appears to improve kinesophobia and functioning overall. Statistical analysis was not provided at time of publishing of preliminary results. Much larger studies in the future are required to determine which protocol will be most efficacious, or if the standard protocol should incorporate a combination of both approaches.

Masoumbeigi et al. (11) published a pilot study with 9 patients with non-specific CLBP looking at pain VAS scores before and 2–4 days after, one-time treatment of 20 Hz rTMS over M1. They found improvement in VAS scores in the treatment group versus baseline of 53%. This study also looked at changes in connectivity between different structures in the brain associated with pain processing, such as: the anterior cingulate (ACC), insula, supramarginal gyrus and prefrontal cortex, using resting state-fMRI. The authors found that after a session of rTMS, connectivity was decreased between the above mentioned brain structures, suggesting a possible mechanism for changes of pain ratings after rTMS in CLBP. One major limitation of this study is that results were only obtained at 2–4 days' post-treatment, it would be interesting to see if changes in connectivity and improvement in pain symptoms were still present weeks after initial treatment.

A study by Nardone et al. (12) describes a benefit of rTMS targeted to the left dorsolateral prefrontal cortex (DLPFC) in pain symptoms of patients with CLBP after spinal cord injury, compared with no change in sham TMS controls. In this small study of 12 subject (*n* = 6 rTMS, *n* = 6 sham), rTMS was applied for 10 sessions over a 2 week period. This study also looked at indices of depression before and after rTMS, finding a significant improvement in depressive symptoms in the treatment group. Interestingly, the improvement in mood developed later in the course of the treatment compared to the improvement of pain symptoms; however, in the follow-up period, pain and mood scores both regressed to pretreatment levels. It should be noted that in this study CLBP was related directly to previous spinal cord injury which is a more specific population compared to other studies looking at CLBP arising from different mechanisms. In the future it would be interesting to see studies addressing other etiology's of CLBP for example mechanical injury not related to the spinal cord, and to compare the results with a similar stimulation protocol.

A study by Lee et al. (13) examined the effects of rTMS on patients with chronic lower back pain (persisting greater than 6 months), with a focus on measuring changes in various psychological symptoms secondary to pain after stimulation of the ACC. The study was completed over 4 weeks (5 days per week, 20 sessions total), with follow up measured 2 weeks after the last treatment session. In this small study (21 subjects) an improvement in depression scores, and a decrease in fear avoidance measures was found with rTMS compared to sham controls. In addition, changes in EEG and heart rate were observed in the treatment group, which brought these measures in line with what is observed in control subjects. Unfortunately, this study did not incorporate a measure of primary pain symptoms and ratings, so it is unclear if rTMS targeting the ACC decreased pain symptoms concurrent with the observed changes in secondary psychological symptoms. This is a major oversight in study design, and would be a worthwhile aspect to explore in further studies. In our review, we also found two case studies presented by Park et al. (14) assessing rTMS in patients with CLBP, specifically looking for improvement in symptoms of depression and insomnia in addition to pain symptoms. They reported improvement in not only subjective pain rating, but also measures of insomnia and depression. In both cases, rTMS was applied over the left prefrontal cortex 5 times per week, for either 3 or 4 weeks. This improvement in non pain symptoms as well as pain symptoms is important to consider when thinking about the application of rTMS to the clinical population. A comparison table of our literature results is presented in Figure 1.

**Figure 1:**
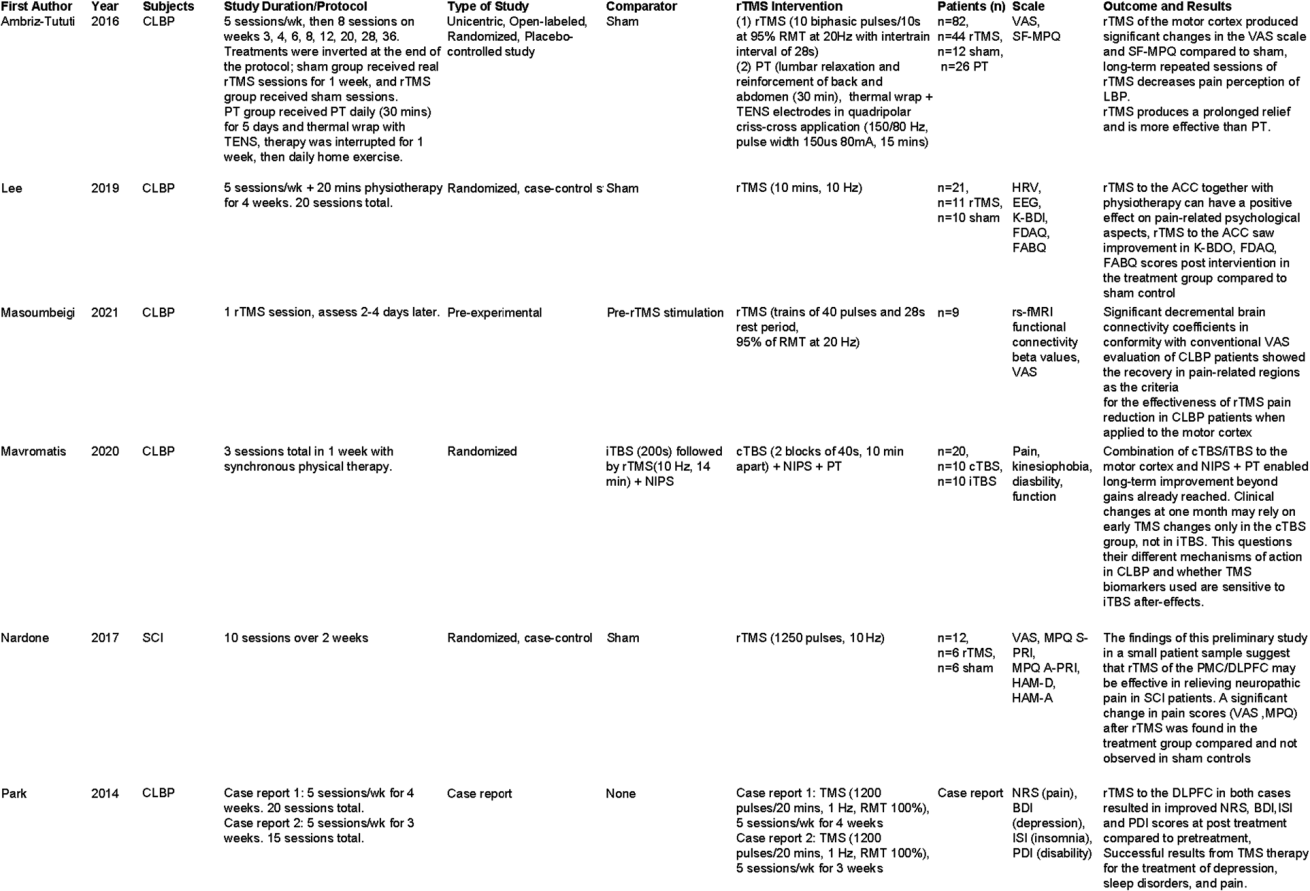
Characteristics of included studies for CLBP and rTM.

As evident from our review of the literature, there continues to be a lack of standardization in studies using rTMS for chronic lower back pain. Questions about the long-term efficacy of rTMS treatment in CLBP remain, as most of the studies we reviewed, aside from Ambriz-Tutui et al. (9), found a loss of treatment effects on pain symptoms in long-term follow up. Future studies should focus on establishing the number and frequency of repeated rTMS treatments required to achieve and maintain a persistent reduction of pain in CLBP. Preliminary findings further suggest that rTMS has the potential to improve other secondary psychological symptoms in patients with chronic pain, including reducing scores for depression and insomnia. Another aspect of a standardized rTMS protocol which remains unclear following our review is the question of which brain region (or regions) should be targeted for maximum beneficial effect. The studies we reviewed variously targeted the ACC, M1, and the left DLPFC. rTMS of M1 and of left DLPFC each reduced pain symptoms, but have not been directly compared, and the effect of targeting the ACC on pain symptoms remains unclear. Furthermore, at least one study variously employed continuous and intermittent rTMS, which alternately may excite or inhibit neural activity in the target region. In each of our studies, the rTMS protocol employed was tolerated without adverse side effects, which adds to its potential clinical suitability as a non- invasive addition to existing chronic lower back pain treatment regimens. A major limitation of the reviewed studies was lack of sample size and lack of active comparators. Future studies to address these issues may employ coordination between multiple clinical research sites and step-wise, sequenced treatment protocols.

One of the issues with rTMS in the CLBP and pain population in general is how this treatment protocol can be applied in a clinic setting. As mentioned above there is currently a gap in the research of large, randomized, controlled blinded studies in the use of rTMS for CLBP. The preliminary studies presented in this review are promising but as pointed out in the review there are issues with design. The use of rTMS in the general is not without issues, since it has been in use for disorders such as depression, there have been questions about the ability to target the correct area of brain tissue based on location of motor cortex elicited from resting motor threshold testing. This is being addressed with developments of more sophisticated methods to predict tissue location based on individual skull size or using MRI imaging data (15, 16). As targeting becomes more specific and controlled for individual differences in brain anatomy the use of rTMS in all applications may see an increase in effectiveness. Another issue with rTMS for the chronic pain population is ease of use, patients with this type of pain often have problems sitting for long periods of time and getting to and from pain clinics. Currently there is portable single pulse TMS machine FDA approved for migraine pain that is portable and able to be used at home by patients (17). Unfortunately, at this time there is no FDA approved machine or protocol for treatment of CLBP or even chronic pain using TMS, although as with the TMS machine approved for migraine this may be something that is developed in the future.

Overall, our review suggests that rTMS may be an effective treatment for pain symptoms in chronic lower back pain across multiple treatment protocols, although there continues to be a lack of standardization and a persistent need for more research including randomized controlled studies, employing larger populations and making a comparison of stimulation targets and parameters to determine the ideal approach to rTMS in this patient population. Evidence from studies reviewed for the indication of CLBP suggest that neuro-navigated targets within the regions M1, DLPFC, and ACC may yield candidate targets for comparison against sham stimulation and each other.

## Author contributions

All authors contributed to the conception, drafting, and editing of this manuscript. All authors contributed to the article and approved the submitted version.

## Conflict of interest

The authors declare that the research was conducted in the absence of any commercial or financial relationships that could be construed as a potential conflict of interest.
